# Efficacy and Safety of the Combination of Palmitoylethanolamide, Superoxide Dismutase, Alpha Lipoic Acid, Vitamins B12, B1, B6, E, Mg, Zn and Nicotinamide for 6 Months in People with Diabetic Neuropathy

**DOI:** 10.3390/nu16183045

**Published:** 2024-09-10

**Authors:** Triantafyllos Didangelos, Eleni Karlafti, Evangelia Kotzakioulafi, Parthena Giannoulaki, Zisis Kontoninas, Anastasia Kontana, Polykarpos Evripidou, Christos Savopoulos, Andreas L. Birkenfeld, Konstantinos Kantartzis

**Affiliations:** 1 Diabetes Center, 1st Propaedeutic Department of Internal Medicine, Aristotle University of Thessaloniki, AHEPA Hospital, 54636 Thessaloniki, Greece; linakarlafti@hotmail.com (E.K.); ekotzaki@auth.gr (E.K.); drziko2401@gmail.com (Z.K.); akontana@auth.gr (A.K.); polysevripidou@gmail.com (P.E.); csavvopo@auth.gr (C.S.); 2 Department of Clinical Nutrition and Dietetics, AHEPA Hospital, 54636 Thessaloniki, Greece; nenagian@yahoo.com; 3 Department of Internal Medicine IV, Division of Endocrinology, Diabetology and Nephrology, University of Tübingen, 72076 Tübingen, Germany; andreas.birkenfeld@med.uni-tuebingen.de (A.L.B.); konstantinos.kantartzis@med.uni-tuebingen.de (K.K.); 4 Institute for Diabetes Research and Metabolic Diseases (IDM), Helmholtz Centre Munich, University of Tübingen, 72076 Tübingen, Germany; 5 German Center for Diabetes Research (DZD), 85764 München-Neuherberg, Germany

**Keywords:** diabetes mellitus type 2, diabetic neuropathy, pain, neuropathic pain, painful diabetic neuropathy, supplements, vitamin supplementation

## Abstract

Aim: To investigate the efficacy of Palmitoylethanolamide (PEA, 300 mg), Superoxide Dismutase (SOD, 70 UI), Alpha Lipoic Acid (ALA, 300 mg), vitamins B6 (1.5 mg), B1 (1.1 mg), B12 (2.5 mcg), E (7.5 mg), nicotinamide (9 mg), and minerals (Mg 30 mg, Zn 2.5 mg) in one tablet in people with Diabetic Neuropathy (DN). Patients–methods: In the present pilot study, 73 people (age 63.0 ± 9.9 years, 37 women) with type 2 Diabetes Mellitus (DMT2) (duration 17.5 ± 7.3 years) and DN were randomly assigned to receive either the combination of ten elements (2 tablets/24 h) in the active group (*n* = 36) or the placebo (*n* = 37) for 6 months. We used the Michigan Neuropathy Screening Instrument Questionnaire and Examination (MNSIQ and MNSIE), measured vibration perception threshold (VPT) with biothesiometer, and Cardiovascular Autonomic Reflex Tests (CARTs). Nerve function was assessed by DPN Check [sural nerve conduction velocity (SNCV) and amplitude (SNAP)]. Sudomotor function was assessed with SUDOSCAN, which measures electrochemical skin conductance in hands and feet (ESCH and ESCF). Pain score (PS) was assessed with Pain DETECT questionnaire. Quality of life was assessed by questionnaire. Results: In the active group, there was a large improvement of pain (PS from 20.9 to 13.9, *p* < 0.001). There was also a significant improvement of vitamin B12 (B12) levels, MNSIQ, SNCV, VPT, and ESCF (222.1 vs. 576.3 pg/ mL, *p* < 0.001; 6.1 vs. 5.9, *p* = 0.017; 28.8 vs. 30.4, *p* = 0.001; 32.1 vs. 26.7, *p* = 0.001; and 72.2 vs. 74.8, *p* < 0.001 respectively). In the placebo group, neither pain (21.6 vs. 21.7, *p* = 0.870) or any other aforementioned parameters changed significantly, and MNSIE worsened (2.9 vs. 3.4, *p* < 0.001). As a result, changes from baseline to follow-up in pain, B12 levels, VPT, and MNSIQ differed significantly between the two groups (*p* < 0.001, 0.025, 0.009, and <0.001, respectively). CARTs, SNAP, ESCH did not significantly change in either of the two groups. Conclusions: The combination of the ten elements in one tablet for 6 months at a daily dose of two tablets in people with DN significantly improves pain, vibration perception threshold, and B12 levels.

## 1. Introduction

Neuropathic pain is one of the most devastating pains and represents a significant unmet medical need. Diabetic Neuropathy (DN) is the leading cause of neuropathic pain [1,2]. Neuropathic pain impairs sleep and significantly impacts the quality of life and the emotional well-being of the patients. This, in turn, predisposes them to depression, noncompliance with treatment, and eventually worsening of all comorbidities. Over the past few decades, considerable advances have been made in diabetic neuropathic pain assessment and management. Several medications are available nowadays, including duloxetine, pregabalin, anticonvulsants, gabapentin, and tricyclic antidepressants. Nevertheless, less than 50% of all patients experience clinically meaningful pain relief (30–50% reduction) [3,4], while the other half remains refractory to these medications [3]. Furthermore, patients frequently experience major adverse effects (drowsiness, dizziness, swelling), leading to non-compliance to treatment. Appropriately targeting current medications and the successful clinical development of drugs acting on new targets remain challenging [2].

Our group recently showed that administration of vitamin B12 (B12) either alone or in combination with other compounds [alpha lipoic acid (ALA), superoxide dismutase (SOD), acetylcarnitine (ALC)] results in a significant clinical improvement in functional aspects (indices) of DN, but only to a moderate and probably slightly clinically significant reduction of neuropathic pain [5,6]. Accordingly, we set out to search for an agent that could more specifically act on neuropathic pain. Such an agent may be palmitoylethanolamide (PEA). The potential analgesic and anti-inflammatory properties of PEA were first reported in 1975 [7], but the first evidence was brought in 1993 by Rita Levi-Montalcini’s group (winner of the Nobel Prize 1986 for the discovery of Nerve Growth Factor [NGF]). The group considered PEA as a ”biological counterpart of the pro-inflammatory NGF”, dose-dependently downregulating hyperactive mast cells via what was thought to be peripheral endocannabinoid receptors at this time [8], and was subsequently proved to be cannabinoid-like G-coupled receptors [9]. There is also some evidence that PEA may have neuroprotective properties by acting via peroxisome proliferator-activated receptor alpha (PPAR-α) receptors [10].

Despite the promising nerve-specific properties, PEA was not used or not even tested in clinical trials until recently, first because patent protection of the pure and natural compound was impossible, and second because of the limited solubility and bioavailability. Recently PEA was micronized (m-PEA) and ultra-micronized (um-PEA), forms that show enhanced rates of dissolution and absorption [11], better bioavailability, pharmacokinetics, and efficacy compared to its naïve form [11,12]. They also lack toxicity and genotoxic potential [11,13]. Nevertheless, up to now, there are very few studies in the literature examining the potential effect of PEA on diabetic neuropathic pain [11,14,15].

Because of these reasons, and following our previous studies, we conducted the present trial to specifically test the efficacy of PEA in the neuropathic pain of diabetic neuropathy. In our country, PEA is not marketed as a single agent, but only in a combination tablet with 9 other elements (PEA 300 mg, SOD 70 UI, ALA, 300 mg, vitamins B6 1.5 mg, B1 1.1 mg, B12 2.5 mcg, E 7.5 mg, nicotinamide 9 mg, and minerals Mg 30 mg and Zn 2.5 mg); we used this easily available formulation for 6 months, a time-frame widely considered to be long enough for any relieving effect on neuropathic pain to be demonstrated [14].

## 2. Materials and Methods

### 2.1. Participants–Eligibility Criteria

Seventy-eight participants were recruited from January 2021 until January 2022 from the outpatient clinic of the Diabetes Center at the University General Hospital of Thessaloniki. Participants were selected in a consecutive manner when they had diabetic neuropathy and manifested pain symptoms. Pain symptoms should be mild to moderate (e.g., tingling, paresthesia, allodynia, dysesthesia) and not require pregabalin or other pain-relieving treatment. Diagnosis of DN was confirmed at baseline with assessments according to the guidelines of the Toronto panel [16]. The diagnosis of DN was set when two or more cardiovascular autonomic reflex tests (CARTs) were abnormal for Diabetic Autonomic Neuropathy (DAN), and when nerve conduction velocity levels and Michigan Neuropathy Screening Instrument Questionnaire (MNSIQ) or Michigan Neuropathy Screening Instrument Examination (MNSIE) were abnormal for Diabetic Peripheral Neuropathy (DPN) [16,17,18,19]. All participants had both DAN and DPN. Patients should have well-controlled glycemia (HbA1c < 7%) [20] for at least 6 months prior to baseline. Participants with uncontrolled diabetes, a recent (within the 6 months prior to baseline) cardiovascular event, chronic kidney disease, a severely advanced form of DN (extreme neuropathic pain or burning feet symptoms that require analgesic medication), and being on analgesic treatment (i.e., antidepressants, antiepileptics, and opioids) and receiving other dietary supplements were excluded. Patients who participated in other clinical studies for diabetic neuropathy treatment in the last 5 years were also excluded.

### 2.2. Randomization and Allocation

We included the first consecutive 78 people with DM2 who fulfilled the eligibility criteria in the study. Three participants were excluded because of severe neuropathic pain and burning feet (were prescribed pregabalin). Two participants changed their address and withdrew their consent before the baseline visit. Finally, 73 patients were randomly allocated to receive the 10-element combination tablet (n = 36) or placebo (n = 37) twice daily for 6 months.

Allocation to treatment is presented in Supplementary Material Figure S1 (CONSORT Flow Diagram). The randomization sequence was generated by the software. Assessors of outcomes and administrators of the supplement, as well as the statistician who performed the analysis, were not aware of the randomization order. Both tablets were designed by the manufacturing company to be similar in appearance and were provided in similar white boxes. Patients were instructed to return the empty containers at follow-up to measure adherence to the treatment. The intervention tablet consisted of SOD, 70 UI or 8.75 mg, PEA, 300 mg, ALA, 300 mg, vitamins B6 (1.5 mg), B1 (1.1 mg), B12 (2.5 μg), E (7.5 mg), nicotinamide, 9 mg, and minerals (Mg 30 mg, Zn 2.5 mg). Dosing instructions were 2 tablets per day in both groups. Patients were advised to take paracetamol in case of pain. Other analgesic medications were not allowed. Participants were advised to not alter their diet and medication regime (including antidiabetic agents) until the end of the study, unless there were complications to their comorbidities. Diet was assessed at baseline and at the end of the 6 months with a 3-day food diary.

### 2.3. Protocol Registration

The study was approved by the Bioethics Committee of Medical School, Aristotle University of Thessaloniki, Greece with number 54230/12.12.2020 and was registered in ClinicalTrials.gov with registration number NCT05984771.

### 2.4. Biochemical Tests

All biochemical parameters (blood count, lipid profile, vitamin B12) were measured with a Cobas-e 602 analyzer with electrochemiluminescence (ECLIA) in the laboratory of University General Hospital of Thessaloniki AHEPA.

### 2.5. Neuropathy Assessment and Tests

All neuropathy assessments were conducted based on the Toronto panel guidelines [16]. Symptoms and clinical signs were evaluated with the Michigan Neuropathy Screening Instrument Questionnaire (MNSIQ) and examination (MNSIE) [19]. For assessing vibration perception, a biothesiometer was used to define the vibration perception threshold (VPT) [21]. Values above 15 V were considered abnormal. [21] Nerve conduction velocity was measured in the sural sensory nerve of both feet with Neurometrix DPN Check and a mean of these values is presented [22]. Pain perception was assessed with the Pain DETECT Questionnaire. A Pain DETECT score between −1 and 38 indicates the likelihood of a neuropathic pain component [23]. Sudomotor function was assessed with SUDOSCAN, which measures electrochemical skin conductance in the feet (ESCF) and hands (ESCH) [24,25,26]. Autonomic neuropathy testing included cardiovascular autonomic reflex tests (CARTs) such as heart rate variability to obtain mean circular resultant (MCR), and the 30:15 stand up test to obtain postural index and blood pressure measurement to test for orthostatic hypotension [18]. Diabetes Quality of Life Questionnaire from the Diabetes Control and Complication Trial was used to assess the quality of life at baseline and at the end of intervention [27].

### 2.6. Statistical Analysis

Sample size was estimated with Gpower 3.1 with 80% power and α set at 0.05. All other statistical analyses were conducted with IBM SPSS v.29 [28]. Continuous variables were checked for normality with a Kolmogorov–Smirnov test and found to be normally distributed. They are presented with mean and standard deviation. Categorical variables are presented with frequencies and percentages. Tests between the 2 groups were conducted with independent Student’s *t* test and from baseline to the end of treatment with paired samples *t*-test. A *p*-value under 0.05 was considered significant [29].

## 3. Results

The baseline characteristics of the participants are presented in Table 1. The study population consisted of overweight or obese elderly subjects (mean age 63.0 ± 9.9 years, mean BMI 31.0 ± 4.2 kg/m^2^). The participants had long-standing DMT2 (mean 17.5 ± 7.3 years) and were taking metformin for a mean of 13.6 years. All patients were relatively well controlled (mean HbA1c 7.0 ± 0.9%). As shown in Table 1, there was no difference between the two treatment groups in terms of anthropometrics and demographic characteristics, diabetes duration, treatment and control, and other co-morbidities at baseline. Diet remained stable throughout the study based on the analysis of food diaries at baseline and follow-up visit.

Common biochemistry did not differ between groups either at baseline or at follow-up, and there was no difference in the change of either parameter between the treatment groups (Table 2). Of particular note, glucose control remained well throughout the study duration, thus excluding better glycemic control as a factor for the favorable effects of the treatment in the active group. Vitamin B12 levels at baseline were 230.3 ± 90.9 pg/mL (167.0 ± 67.1 pmol/L), indicating inadequate supply [30,31]. Nevertheless, vitamin B12 deficiency was rather “functional” [30] because none of the patients had anemia, and not even the MCV of the patients was out of the normal range. After 6 months of treatment, vitamin B12 levels, as expected, significantly increased in the active group and reached levels that are considered to be adequately high, both according to WHO recommendations and according to recommendations for elderly people in particular. In the placebo group, vitamin B12 levels also increased, but not significantly, remaining in the range considered to be the “grey zone”, particularly for elderly people (Table 2).

Baseline neuropathy assessment results are presented in Table 3. All participants presented symptoms of diabetic neuropathy already at baseline and had been diagnosed with peripheral and painful diabetic neuropathy. The vibration perception threshold was impaired significantly, as well as the sural nerve conduction velocity. Of particular importance, there was no difference in any of the results of the neuropathy tests between the active and the placebo-treated groups at baseline (Table 3).

During the follow-up, there was a small but significant improvement in SNCV, a clearly significant improvement in VPT, and the pain score in the active group, whereas none of these indices improved in the placebo group (Table 4). Among the other test results, MNSIQ improved significantly in the active group, probably because it includes questions about pain. No other neuropathy indices changed significantly. Of note, this improvement cannot be attributed to respective changes in glycemic control, because the latter remained fair, good, and stable during the follow-up in both arms. There was a deterioration in MNSIE in the placebo group, suggesting that more stringent glycemic control is required to protect against the progression of DN and that the study medication protected the participants in the active group from deterioration of their DN.

Because of the great variability in the improvement of the pain score even in the active group (ranging from −16 to +2), the question arises whether the patients who experienced the largest (or vice versa, the lowest) improvement in pain already had any particular characteristics at baseline. To answer the question, we divided the subjects of the active group in tertiles of pain score improvement and compared the baseline characteristics of the participants between the tertiles (Supplementary Material, Table S1). Patients in the upper tertile (who experienced the largest improvement of pain) had significantly higher baseline pain scores than those in the lowest tertile (p ANOVA 0.007, p post hoc upper vs. lowest tertile 0.015), meaning that patients with more severe pain perceived more benefit from the study medication. This is most probably due to a bottom effect in patients in the lowest tertile. Another putative (but less possible) explanation is that patients with severe pain subjectively perceived any improvement of pain as being particularly “great” and rated it as such in the questionnaires. No other baseline characteristic differed between the 3 tertiles. However, it should be kept in mind that the number of subjects in each group (n = 12) is rather small.

## 4. Discussion

We designed and conducted the present study to extend the understanding and the clinical utility of the findings of two recent studies of our group, in which we showed that administration of vitamin B12, either alone or in combination with other compounds (ALA, SOD, and ALC), leads to a significant improvement in functional aspects (indices) of DN, but to only a moderate improvement in the diabetic neuropathic pain score [5,6]. Accordingly, in this study, we aimed primarily at a reduction in the pain score and set out to test the combination of vitamin B12 with PEA, a compound with potential anti-inflammatory and analgesic properties, which, notably, may be particularly specific for the nervous system [13,15,32,33,34]. Since there is only one approved formulation containing PEA in our country, we had to use a tablet containing not only PEA and vitamin B12, but also SOD, ALA, vitamins B1, B6, nicotinamide, and the trace elements Mg and Zn. We administered the tablet twice a day for 6 months, not for one year as in our previous studies [5,6], because it is widely accepted that 6 months is enough for an effective agent to show its neuropathic pain-relieving potency (while any improvement in nerve functionality may need more time to be achieved) [35].

We found an impressively, almost unexpectedly, large reduction in the pain score (by 33%) in the active group. Such a magnitude of improvement of the neuropathic pain is comparable with the efficacy of pain of antiepileptics and anti-depressive drugs [36]. In the placebo group, the pain score remained substantially unchanged. A reduction in pain scores was observed in 27 out of 36 patients (75%) in the active group and 5 out of 37 patients (14%) in the placebo group, most probably due to a placebo effect. Consistent with the reduction in the pain score, QL and MNSIQ (which contains at least 2 questions about neuropathic pain) also significantly improved in the active group, while, again, remaining substantially unchanged in the placebo group. The clear (>10%) improvement in QL strongly suggests that the reduction in the pain score in the active group is not only large, but also clinically significant. This is because the pain at baseline, with a mean score of 21, could have been considered to be mild to moderate. If that was the case, the QL (and the MNSIQ) would show only a little improvement (or none at all) during the follow-up.

The marked analgesic effect of the trial medication has to be most likely attributed to PEA (or to a synergistic effect of PEA with other ingredients of the tablet). In a previous study of our group in a similar population, administration of a much higher vitamin B12 dose (1000 μg daily vs. 5 μg daily in the present study) for 1 year (vs. 6 months in the present trial) resulted in higher vitamin B12 levels, but only in a minimal reduction in pain scores [6]. In another study, again in a similar population, a combination of higher daily doses of vitamin B12 (250 μg), SOD (10 mg vs. 8.75 mg in the present study), and ALA (570 mg vs. 300 mg in this study) for 1 year resulted in a very moderate (7%) reduction in pain score (from a mean of 19 to a mean of 17) [5]. It follows that the contribution of vitamin B12, SOD, or ALA to the improvement of the pain shown in the present trial must be rather small. In theory, the other vitamins of the B complex that the study medication tablet contained may have had a synergistic effect with ALA and/or SOD on neuropathic pain [37,38,39]. However, this combination was previously shown to have a beneficial effect mainly on nerve conduction velocity, i.e., on nerve functionality [37,38,39], not in pain. In the literature, there is some, albeit not very robust, evidence that the combination of nicotinamide, the amide form of the vitamin B3 (niacin), with magnesium may exert some analgesic effect. Administration of nicotinamide in a range of 5.0–25.0 mmol/L can significantly protect neurons during oxidative stress injuries and apoptosis. Magnesium exerts an antinociceptive action by antagonizing the action of the N-methyl-d-aspartate (NDMA) receptor, preventing central sensitization, and attenuating pain hypersensitivity. Brain magnesium levels are reduced in both depression and chronic pain [40], whereas supplementation with Mg has shown promising results in alleviating neuropathic pain not only in diabetic neuropathy but also in other neuropathic conditions [13,14,15,40,41,42]. Taken together, the other vitamins of the B complex and minerals in the study medication may act as catalysts or co-enzymes, but are not likely to have analgesic effects on their own, and PEA is probably responsible for the largest part of the observed pain relief. Of note, specific analgesic medication was not allowed during the study, and only 3 or 4 patients in every group took occasionally paracetamol for a few days.

Although it is generally accepted that PEA is an endogenous fatty acid amide that is produced on demand and acts locally at the site of injury and tenderness to reduce inflammation and pain, the precise mechanisms by which PEA exerts its anti-inflammatory and analgesic effects are not known. Available evidence suggests that PEA binds with high affinity to at least two kinds of receptors, the membrane cannabinoid-like G-Protein Coupled receptors GPR 55 and GPR 119, and the nuclear receptor peroxisome proliferator-activated receptor alpha (PPAR-α) [34]. The anti-inflammatory effect is probably mediated by both receptors, since PEA was shown to downregulate hyperactive mast cells through cannabinoid-like G-coupled receptors in a dose-dependent manner and to downregulate macrophage infiltration via PPAR-a activation [43]. Studies in PPAR-α null mice showed that activation of PPAR-α is necessary for the analgesic action of PEA [14]. This analgesic effect seems to be exerted both in the peripheral and the central nervous system. Two mechanisms have been described: a rapid analgesic effect that involves activation of calcium-dependent potassium channels, and a slower effect, which is comprised of neurosteroid de novo synthesis [44] and increased allopregnanolone (a 3α,5α-progesterone metabolite) production [44], ultimately leading to allosteric modulation of γ-aminobutyric acid (GABA) type A receptors [44]. PEA cannot directly bind to cannabinoid receptors 1 and 2 (CB1 and CB2). Nevertheless, PEA may indirectly activate the endocannabinoid system by inhibiting the degradation of the endocannabinoid anandamide, a phenomenon known as the “entourage effect” [34]. It has also been suggested that PEA-induced PPAR-α activation can increase CB2 mRNA and protein expression [45]. Finally, an action of PEA on the transient receptor potential vanilloid receptor 1 (TRPV1) channels has also been proposed. This action is supposed to contribute to the significant anti-nociceptive effect of PEA [46].

Evidence from the use of PEA in reducing pain sensation comes from five clinical studies and one RCT [11,12,14,37,43,47]. PEA administration resulted in an approximate 30–40% reduction of pain in all of these studies. Furthermore, taking all studies together, it can be concluded that PEA is effective in managing neuropathic pain as early as after only 10 days of administration. There is some evidence that PEA specifically reduces the pain characteristic of paresthesia/dysesthesia [15].

In addition to the great reduction of pain in the active group, we found a significant improvement in some of the indices of nerve functionality, such as NCV, VPT, and ESCF. These results are in line with the findings of our previous studies, in which administration of B12 alone or in combination with SOD, ALA, and carnitine in a similar population with diabetic neuropathy resulted in a significant improvement of nearly all indices of nerve function [5,6]. In the present study, the magnitude of the improvement of VPT and NCV was somehow smaller than in the previous studies. This fact, as well as the absence of a significant effect on SNAP in the present study, may be explained by the longer duration of the previous studies (12 months) in comparison to the 6 months in this study. In other studies, the time needed to observe a difference (either improvement or deterioration) in functional neuropathy indices was at least 1 year [48,49]. Since the metabolic control was stable (HbA1c ~7.0%) in both groups during the 6 months of follow-up, and antidiabetic, antihypertensive, and hypolipidemic medication did not change, the improvement in the neurophysiological parameters should be probably attributed, as in the previous studies of our group, to the remaining contents of the study medication, most probably vitamin B12, ALA, and SOD, or a synergistic effect of the latter two with the other vitamins of the B complex, Mg, and Zn.

With regard to ALA, in our hands, it did not seem to have a strong pain-relieving effect, but rather a favorable effect on nerve functionality [5]. This is not quite in line with existing data in the literature; the vast majority of the several studies in which ALA was tested demonstrate an analgesic effect of ALA in diabetic painful neuropathy. The primary outcome of most of these studies was an improvement in the total symptom score [37,38,39,50,51,52,53]. Only a few trials suggest that ALA may improve neurophysiological parameters in DN [38,39,52,54]. A putative explanation may be that ALA needs the synergistic action of other compounds, such as vitamins of the B complex in the present study. The mechanism of action of ALA is largely unknown, but it has been repeatedly shown to improve glucose homeostasis and lipid profile (except HDL cholesterol) [37,38,39,50,51,52,53].

Unlike ALA, clinical studies with SOD have shown an improvement in both neurophysiological indices and in symptoms (including pain) of diabetic neuropathy [5,38]. Also, experimental data show an association between low levels of SOD and the progression of all aspects of DN [55]. SOD is a metalloenzyme, which has a potent antioxidant action by neutralizing superoxide that is overproduced due to hyperglycemia. This oxidative stress is considered one of the main factors contributing to the pathogenesis of diabetic neuropathy. It is therefore why the combination of ALA with SOD is believed to act at more than one site inhibiting the pathogenetic mechanisms of diabetic neuropathy and not only as a symptomatic, pain-relieving treatment.

The combinations of vitamins of the B complex (B12, B1, B6) were tested in six studies. In four of them, the combination showed a clear beneficial effect on nerve conduction velocity [5,6,56,57]; this is shown also in a meta-analysis by Stein et al., 2021 [57]. The majority of studies with the combination of vitamins of the B complex with other compounds demonstrated a favorable effect on neuropathic pain. In these studies, there seems to be an apparent synergetic effect of vitamins of the B group (B1, B6, B12, and folic acid) [5,6,56,57] with other bioactive compounds such as ALA, SOD, and ALC [5,57]. Moreover, the combination of vitamins of the B group and analgesic medications, such as pregabalin and gabapentin, appear to have an additive action effect on neuropathic pain [56].

Data on vitamin E and its use in DN are controversial [58]. However, in recent studies, there are some promising results that vitamin E may improve nerve conduction velocity in individuals with DM2 [59]. Zinc deficiency was shown to have an association with diabetic neuropathy and the degree of its severity in terms of nerve conduction velocity and neuropathic symptoms [60].

Το our knowledge, our study is unique in that we used this 10-combination tablet for treating mild to moderate symptoms of painful diabetic neuropathy. The combination of PEA with the nine compounds [SOD, ALA, vitamins B6, B1, B12, E, nicotinamide, and minerals (Mg, Zn)] was found to very effectively decrease pain in mild to moderate PDN with no actual side effects at all. Of particular note, the patients in our study had good glycemic control at baseline and remained well-controlled throughout the trial. Thus, the entire favorable effect on pain (and the other markers of neuropathy) can be attributed solely to the investigated medication. Another strength of our study is that, although the main outcome was the reduction in pain, we included patients presenting both forms of diabetic neuropathy (peripheral and autonomic) and neuropathic pain in the trial, and, unlike most other studies testing a possible effect on pain, we measured the efficacy of the investigational drug, mainly PEA, on all parameters of DN, including neurophysiological measurements and autonomic nervous system function.

We also have to acknowledge certain limitations of our study. The treatment arms consisted of a rather small absolute number of patients and the duration of the study was also rather short, 6 months. However, according to the power analysis, the sample was not too small. Furthermore, the 6 months’ follow-up is considered to be enough for any effect of a drug on neuropathic pain to be demonstrated [14]. All patients had mild to moderate neuropathic pain and came from only one diabetes center. Therefore, the findings of this study must be confirmed in larger randomized controlled trials including other populations and also patients with more severe neuropathic pain. Finally, the 10 ingredients of the study tablet do not allow for determining the separate effect of each of them. However, we could also consider the possible synergistic effects of more than one compound. In any case, as discussed above, the prominent role of PEA, alone or not, in relieving pain should not be questioned.

## 5. Conclusions

The combination of the ten elements in one tablet for 6 months at a daily dose of two tablets in people with diabetic neuropathy significantly improved neuropathic pain. It also increased vitamin B12 levels, and, despite the somehow short follow-up duration, improved some aspects of nerve functionality, such as vibration perception threshold. Our findings suggest that PEA can be administered for an extended period without significant side effects and may be helpful in alleviating the mild to moderate neuropathic pain of diabetic neuropathy. In more severe neuropathic pain, PEA may be a valuable adjunct to other analgesic drugs in order to enhance the effectiveness or to prevent the necessity of using increased doses of analgesics that could lead to adverse events.

## Figures and Tables

**Table 1:** Baseline characteristics of the study population.

	All	Active Group	Placebo Group	*p*
Gender (m/w)	36/37	16/20	20/17	0.204
Age (years)	63.0 ± 9.9	64.5 ± 10.8	61.5 ± 8.9	0.243
Height (cm)	166 ± 9	165 ± 9	167 ± 9	0.393
Body weight (kg)	85.4 ± 9.2	84.0 ± 10.2	86.7 ± 8.0	0.251
BMI (kg/m^2^)	31.0 ± 4.2	30.9 ± 4.3	31.2 ± 4.1	0.778
Diabetes duration (years)	17.5 ± 7.3	17.9 ± 8.3	17.1 ± 6.2	0.632
Only OADs, incl. metformin (n,%)	45 (61.6%)	21 (58.3%)	24 (64.9%)	0.667
OADs and basal insulin (n,%)	28 (38.4%)	15 (41.7%)	13 (35.1%)	0.568
Smoking (n,%)	15 (22%)	7 (20%)	8 (24.2%)	0.110
Cardiovascular Disease (n,%)	28 (41.2%)	15 (42.8%)	13 (39.4%)	0.587
Dyslipidemia (n,%)	52 (76.5%)	28 (80%)	24 (72.7%)	0.853
Hypertension (n,%)	54 (79.4%)	26 (74.3%)	28 (84.8%)	0.781
HbA1c (%)	7.0 ± 0.9	7.0 ± 0.9	7.1 ± 0.8	0.817
Vitamin B12 (pg/mL)	230.3 ± 90.9	222.1 ± 87.5	238.3 ± 94.9	0.493

Abbreviations: OADs: oral antidiabetic drugs. Cardiovascular disease is defined as past (older than 6 months before baseline) stroke, atrial fibrillation, cerebrovascular disease, or myocardial infraction.

**Table 2:** Biochemical/Laboratory markers.

	All	Active Group (n = 36)			Placebo Group (n = 37)			*p* of Change
		Baseline	Follow Up	*p*	Baseline	Follow Up	*p*	
Total Cholesterol (mg/dL)	162.3 ± 37	158.06 ± 32.8	159.2 ± 30.8	0.229	166.7 ± 41.1	165.5 ± 40.7	0.282	0.339
HDL Cholesterol (mg/dL)	47.7 ± 11.7	46.9 ± 12.8	46.7 ± 12.7	0.213	48.6 ± 10.5	48.8 ± 10.4	0.160	0.472
LDL cholesterol (mg/dL)	86.8 ± 28.9	84.3 + 30.6	85.7 ± 28.9	0.243	89.3 ± 30.8	87.9 ± 29.7	0.756	0.345
Triglycerides (mg/dL)	139.3 ± 43	134.4 ± 39.9	133.5 ± 38.5	0.593	144.5 ± 46.1	144.3 ± 44.6	0.891	0.339
TSH	2.3 ± 0.8	2.4 ± 0.7	2.4 ± 0.7	0.247	2.3 ± 0.8	2.3 ± 0.8	0.160	0.09
AST	18.9 ± 8	20.1 ± 9.9	19.9 ± 9.8	0.481	17.5 ± 5.2	17.4 ± 5.2	0.484	0.192
ALT	21.9 ± 10.1	21.9 ± 11.5	21.8 ± 11.3	0.912	22 ± 8.6	21.7 ± 7.8	0.299	0.477
Urea (mg/dL)	34.2 ± 11.5	33.4 ± 9.2	33.4 ± 9.2	0.737	35.1 ± 13.5	34.9 ± 13.6	0.563	0.279
Creatinine (mg/dL)	0.9 ± 0.2	0.9 ± 0.2	0.9 ± 0.2	0.612	0.9 ± 0.2	0.8 ± 0.2	0.326	0.365
Ht (%)	40.5 ± 2.9	40.7 ± 2.9	40.6 ± 2.8	0.454	40.3 ± 2.9	40.4 ± 2.8	0.694	0.323
MCV	85.5 ± 3.9	84.9 ± 4	86.2 ± 3.6	0.342	86.2 ± 3.6	86 ± 3.5	0.492	0.177
WBC	7.9 ± 1.4	7.9 ± 1.5	7.9 ± 1.4	0.424	7.9 ± 1.4	7.9 ± 1.3	0.560	0.894
PLT	239.1 ± 5.7	246.1 ± 41	249 ± 40.7	0.159	231.8 ± 58.9	233.7 ± 58.8	0.188	0.246
HbA1c (%)	7.0 ± 0.9	7.0 ± 0.9	6.83 ± 0.3	0.289	7.1 ± 0.8	6.9 ± 0.3	0.341	0.749
B12 (pg/mL)	230.3 ± 90.9	222.1 ± 87.5	576.3 ± 201.5	**<0.001**	238.3 ± 94.9	264.3 ± 106.1	0.081	**<0.001**

Abbreviations: HDL: high-density lipoprotein cholesterol, LDL: low-density lipoprotein cholesterol, TSH: thyroid stimulating hormone, AST: aspartate aminotransferase ALT: alanine transaminase Ht: Hematocrit, MCV: mean corpuscular volume WBC: white blood count, PLT: platelets HbA1c: Glycated Hemoglobin, B12: Vitamin B12. Bold indicates the significant results (*p* < 0.05).

**Table 3:** Results of Neuropathy Assessment Tests at baseline.

	All	Active Group (n = 36)	Placebo Group (n = 37)	*p*
MNSIQ	6.2 ± 1.7	6.13 ± 1.9	6.3 ± 1.5	0.779
MNSIE	3.3 ± 2.2	3.6 ± 1.9	2.9 ± 2.4	0.241
Pain score	20.4 ± 7.7	20.9 ± 7.4	21.6 ± 7.9	0.224
SNAP (μV)	5.9 ± 4.1	6.5 ± 3.8	5.4 ± 4.4	0.281
SNCV (m/s)	29.4 ± 22.6	28.8 ± 22.7	30 ± 22.9	0.831
VPT (V)	30.6 ± 14.5	32.1 ± 14.5	29.2 ± 14.5	0.437
MCR	20 ± 30.9	18.4 ± 34.6	21.8 ± 27.8	0.789
PI	4.3 ± 6.6	5.9 ± 8.9	2.5 ± 0.9	0.200
PO (mmHg)	8.9 ± 11.8	10.7 ± 12.8	7.1 ± 10.8	0.456
ESCF (μS)	76.9 ± 11.5	72.2 ± 10.7	75.6 ± 11.7	0.775
ESCH (μS)	68 ± 10.5	68.4 ± 11.1	67.6 ± 10.2	0.764

Abbreviations: MNSIQ: Michigan Neuropathy Screening Instrument Questionnaire, MNSIE: Michigan Neuropathy Screening Instrument Examination, SNAP: Sural Nerve Action Potential (amplitude), SNCV: Sural sensory Nerve Conduction Velocity, VPT: Vibration Perception Threshold, MCR: Mean Circular Resultant, PI: Postural Index (postural hypotension), PO: Orthostatic Hypotension, ESCF: Electrochemical Skin Conductance in Feet, ESCH: Electrochemical Skin Conductance in Hands.

**Table 4:** Changes in neuropathy indices from baseline to follow-up in both treatment arms.

	Active Group (n = 36)			Placebo Group (n = 37)			*p* for Difference between Groups
	Baseline	Follow Up	*p*	Baseline	Follow Up	*p*	
HbA1c (%)	7 ± 0.9	6.83 ± 0.3	0.289	7.1 ± 0.8	6.9 ± 0.3	0.341	0.749
B12 (pg/mL)	222.1 ± 87.5	576.3 ± 201.5	**<0.001**	238.3 ± 94.9	264.3 ± 106.1	0.081	**<0.001**
MNSIQ	6.13 ± 1.9	5.9 ± 1.8	**0.017**	6.3 ± 1.5	6.23 ± 1.5	0.712	0.117
MNSIE	3.6 ± 1.9	3.7 ± 1.8	0.161	2.9 ± 2.4	3.4 ± 2.4	**<0.001**	**0.009**
Pain score	20.9 ± 7.1	13.9 ± 6.1	**<0.001**	21.6 ± 7.9	21.7 ± 8.4	0.870	**<0.001**
Quality of life	38.3 ± 11.6	35.6 ± 10.3	**<0.001**	38.3 ± 10.4	38.8 ± 9.7	0.208	0.779
SNAP (μV)	6.5 ± 3.8	7.2 ± 3.6	0.055	5.4 ± 4.4	5.4 ± 4.3	0.897	0.101
SNCV (m/s)	28.8 ± 22.7	30.4 ± 23.04	**0.001**	30 ± 22.9	29.4 ± 23.5	0.564	0.052
VPT (V)	32.1 ± 14.5	26.7 ± 12.9	**0.001**	29.2 ± 14.5	28.5 ± 13.7	0.618	**0.025**
MCR	18.4 ± 34.6	14.6 ± 15.3	0.686	21.8 ± 27.8	17.5 ± 28.3	0.695	0.951
PI	5.9 ± 8.9	5.7 ± 9.7	0.833	2.5 ± 0.9	1.9 ± 1.3	0.195	0.946
PO (mmHg)	10.7 ± 12.8	5.5 ± 9.1	0.211	7.1 ± 10.8	6.3 ± 9.8	0.765	0.377
Valsalva	1.5 ± 0.2	1.6 ± 0.2	0.883	1.5 ± 0.3	1.5 ± 0.2	0.963	0.971
ESCF (μS)	72.2 ± 10.7	74.8 ± 10.8	**<0.001**	75.6 ± 11.7	75.8 ± 11.6	0.731	0.239
ESCH (μS)	68.4 ± 11.1	69.2 ± 10.5	0.567	67.6 ± 10.2	68.5 ± 8.9	0.467	0.937

Abbreviations: MNSIQ: Michigan Neuropathy Screening Instrument Questionnaire, MNSIE: Michigan Neuropathy Screening Instrument Examination, SNAP: Sural Nerve Action Potential (Amplitude), SNCV: Sural sensory Nerve Conduction Velocity, VPT: Vibration Perception Threshold, MCR: Mean Circular Resultant, PI: Postural Index (postural hypotension), PO: Orthostatic Hypotension, ESCF: Electrochemical Skin Conductance in Feet, ESCH: Electrochemical Skin Conductance in Hands. Bold indicates the significant results (*p* < 0.05).

## Data Availability

Data is available upon request.

## Supplementary Materials

The following supporting information can be downloaded at: https://www.mdpi.com/article/10.3390/nu16183045/s1, Figure S1: CONSORT Flow Diagram; Table S1: Comparison of baseline characteristics of participants in the active group between tertiles of pain score improvement.
