# Supplementary material for: Efficacy and Safety of the Combination of Palmitoylethanolamide, Superoxide Dismutase, Alpha Lipoic Acid, Vitamins B12, B1, B6, E, Mg, Zn and Nicotinamide for 6 Months in People with Diabetic Neuropathy
Source: Nutrients. 2024 Sep 10;16(18):3045. doi: 10.3390/nu16183045 (PMC11434759; doi:10.3390/nu16183045)
Supplement: Supplementary file 1 [file nutrients-16-03045-s001.zip › nutrients-3140636-supplementary.pdf]

Efficacy and Safety of the combination of Palmitoylethanolamide, Superoxide Dismutase, Alpha Lipoic Acid, Vitamins B12, B1, B6, E, Mg, Zn and Nicotinamide for 6 months in people with diabetic neuropathy

Triantafyllos Didangelos, Eleni Karlafti, Evangelia Kotzakioulafi, Parthena Giannoulaki, Zisis Kontoninas, Anastasia Kontana, Polykarpos Evripidou, Christos Savopoulos, and Konstantinos Kantartzis

Supplementary Material

Figure S1. CONSORT Flow Diagram

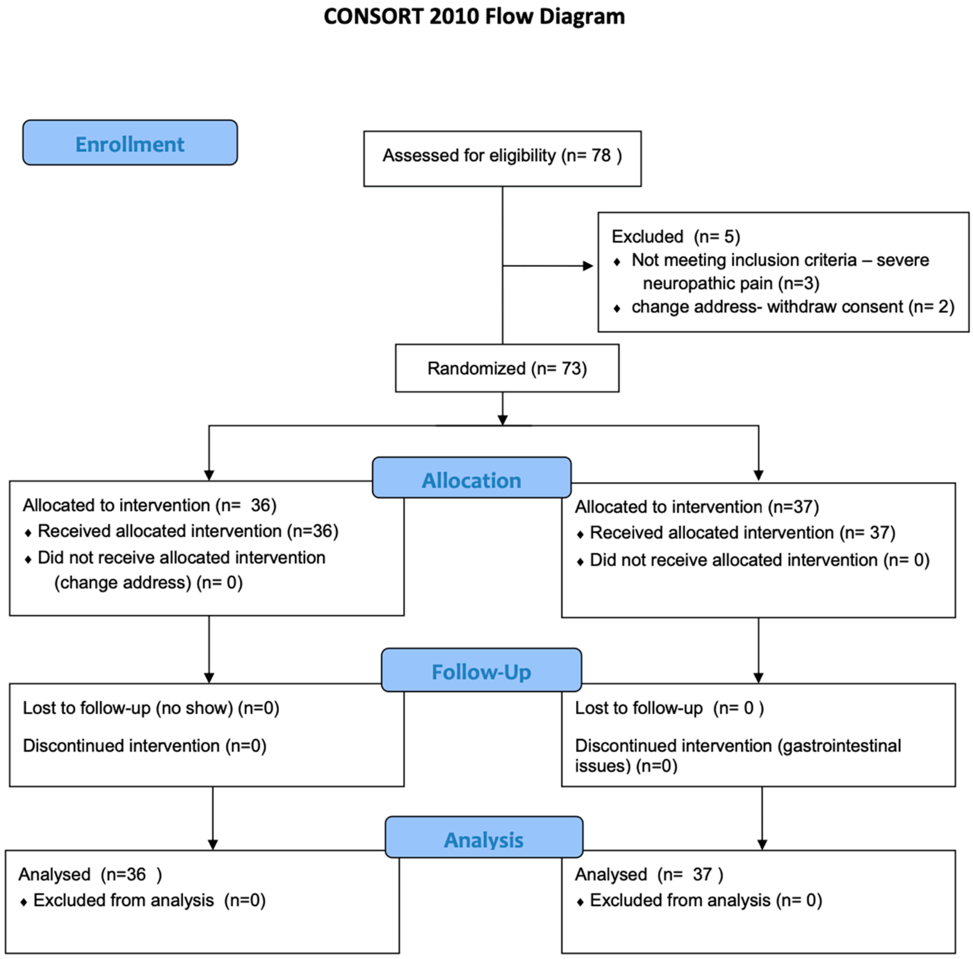

Supplementary Table S1. Comparison of baseline characteristics of participants in the active group between tertiles of pain score improvement

**Characteristic**	**Upper tertile of pain score improvement (n=12)**	**Medium tertile (n=12)**	**Lowest tertile of pain score improvement (n=12)**	**p Anova**	**p post hoc Upper vs lowest tertile**
Age (y)	64.5 ± 9.6	65.1 ± 11.8	64.1 ± 12.3	0.981	1
Weight (kg)	83.6 ± 7.9	85.8 ± 12.4	82.9 ± 11.1	0.827	1
Diabetes duration (y)	20.3 ± 9.8	15.2 ± 7.7	17.9 ± 7.1	0.414	1
Metformin duration (y)	18 ± 11.1	13.6 ± 8.3	13 ± 7.4	0.403	0.667
HbA1c (%)	6.9 ± 1	6.9 ± 0.8	7.1 ± 0.9	0.903	1
Ht (%)	39.5 ± 2.2	39.3 ± 3.7	39.5 ± 2.7	0.920	1
MCV (fL)	88.2 ± 5	85.7 ± 4.5	86.8 ± 4.5	0.462	0.469
Vit. B_{12} (pg/ml)	201.8 ± 60.3	272.9 ± 108.7	198.8 ± 80.3	0.113	1
MNSIQ	6.4 ± 1.9	5.3 ± 2.1	6.6 ± 1.6	0.313	1
MNSIE	3.5 ± 2	3.3 ± 2	3.9 ± 1.8	0.813	1
SNAP (lV)	6.5 ± 4.1	7.4 ± 4.3	5.7 ± 3.3	0.626	1
SNCV (m/s)	19.7 ± 21.3	29.7 ± 21.9	38.9 ± 22.9	0.172	0.191
VPT (V)	34.4 ±14.9	28.2 ± 13.3	33 ± 15.8	0.639	1
Pain Score	26 ± 5.6	18.1 ± 6.2	17.8 ± 6.6	**0.007**	**0.015**
MCR	12.9 ± 10.3	8.4 ± 8.1	7.3 ± 4.8	0.261	0.373
Postural Index	2.7 ± 0.5	8 ± 11.6	2.4 ± 0.7	0.115	1
Postural Hypotension (mm/Hg)	6.5 ± 11.1	10.3 ± 13.4	17 ± 13.1	0.169	0.190
ESC Feet (μS)	77.1 ± 7.4	70.8 ± 12.6	68.1 ± 10.7	0.140	0.167
ESC Hands (μS)	67.1 ± 10.9	71.3 ± 9.7	67.3 ± 12.9	0.657	1
